# Adjustment of impact phenolic compounds, antioxidant activity and aroma profile in Cabernet Sauvignon wine by mixed fermentation of *Pichia kudriavzevii* and *Saccharomyces cerevisiae*

**DOI:** 10.1016/j.fochx.2023.100685

**Published:** 2023-04-19

**Authors:** Wenhan Liu, Rujie Ji, Ayinuer Aimaier, Jinkui Sun, Xilei Pu, Xuewei Shi, Weidong Cheng, Bin Wang

**Affiliations:** College of Food Science and Technology, Shihezi University, Shihezi 832000, China

**Keywords:** *Pichia kudriavzevii*, Mixed fermentation, Antioxidant activity, Polyphenolics, Aroma

## Abstract

Mixed fermentation using saccharomyces cerevisiae and non-*saccharomyces cerevisiae* has become one of the main research strategies to improve wine aroma. Hence, this study applied the mixed fermentation technique using *Pichia kudriavzevii* and *Saccharomyces cerevisiae* to brew Cabernet Sauvignon wine and to investigate the effects of inoculation timing and inoculation ratio on the polyphenolics, antioxidant activity and aroma of the resulting wine. The results showed that mixed fermentation significantly improved the amounts of flavan-3-ols. In particular, S1:5 had the highest amounts of (-)-catechin and procyanidin B1 (73.23 mg/L and 46.59 mg/L), while S1:10 had the highest (-)-epicatechin content (57.95 mg/L). Meanwhile, S1:10 showed the strongest FRAP, CUPRAC and ABTS + activities (31.46 %, 25.38 % and 13.87 % higher than that of CK, respectively). In addition, mixed fermentation also increased the amounts of phenylethanol, isoamyl alcohol and ethyl esters, which enhanced the rose-like and fruity flavor of wine. This work used a friendly non-*saccharomyces cerevisiae* alongside appropriate inoculation strategies to provide an alternative approach for improved wine aroma and phenolic profile.

## Highlights

• Sequential inoculation and co-fermentation affect the quality of wine from different levels.
• Mixed fermentation of *P.kudriavzevii* and *S.cerevisiae* improve rose and fruity flavor by accumulating phenylethyl alcohol, isoamyl alcohol and ethyl ester.
• Sequential inoculation has a significant contribution to the nutritional value of wine.
• Sequential inoculation and co-fermentation are feasible solutions to solve the homogenization of wine.

## Introduction

As a result of the warming climate and the widespread application of commercial inoculation, wine styles have gradually become more homogeneous. In particular, the widespread use of commercial yeasts, which is not a good display of the unique style of wine. Therefore, improving the flavor of wine by optimizing fermentation has gradually attracted attention. For instance, low temperature fermentation could increase the ethanol, ethyl acetate and ethyl butanoate content (Du, Ye, Zang, Nan, & Liu, 2022). I However, more recently, the use of mixed fermentation involving *Saccharomyces cerevisiae* and non-*Saccharomyces cerevisiae* species has emerged as a powerful means to change the results of wine fermentation.

With increasing research on non-*Saccharomyces cerevisiae*, the latter’s biodiversity and brewing characteristics have gradually attracted attention in the wine industry. Indeed, studies have shown that wines produced by wild yeasts in major wine-producing countries around the world were incomparable in terms of their complexity, structure, aroma and regional characteristics, etc (Rwnouf, Claisse, & Lonvaud, 2005). During the early stages of the fermentation process, *Saccharomyces cerevisiae* needs to gradually adapt to the new environment, and since at this point it is usually in a lag phase, non-*Saccharomyces cerevisiae* tend to be the dominant flora (Rojas, Gil, Piñaga, & Manzanares, 2001). However, as fermentation progresses, the alcohol content in grape mash increases and the oxygen content decreases. As a result, most aerobic non-*Saccharomyces cerevisiae* and non-*Saccharomyces cerevisiae* with a weak tolerance to ethanol gradually decline in numbers. At the same time, with an increasing population of *Saccharomyces cerevisiae*, competitive inhibition also become a main reason for the decrease of non-*Saccharomyces cerevisiae* cells (Varela & Borneman, 2017). However, there are some yeasts, such as *Hansenula sporogenes* and *Metschnikowia pulcherrima*, which have the ability to produce alcohol. Similarly, *Paecilomyces abdominis* can survive in a high-alcohol environment (12% (v/v)) (Clavijo, Calderón, & Paneque, 2010). Aroma is an important indicator that plays a key role in distinguishing between different wines. In recent years, many studies have shown that mixed fermentation using *Saccharomyces cerevisiae* and non-*Saccharomyces cerevisiae* could actually improve the content of aromatic compounds in wine (Wang, Li, Dizy, Ullah, Sun, & Tao, 2017). In particular, Renault et al. (Renault, Coulon, Reve, Barbe, & Bely, 2015) showed that mixed fermentation with *Saccharomyces cerevisiae* and *Torulaspora delbrueckii*, through co-inoculation and sequential inoculation,increased the amounts of special esters such as ethyl propionate and ethyl isobutyrate. Similarly, Zhao et al. (Zhao et al., 2022) indicated that, sequential inoculum improved wine aroma, including yeast-derived and grape-derived volatiles. In addition, *Pichia fermentans*, either dead or alive, promoted the growth of *Oenococcus oeni*, thereby suggesting that it could be a potential candidate for inducing dependable malolactic fermentation of wine.

As people started to pay more attention to dietary health, the positive impact of wine on human health has also been largely studied. To date, >1600 compounds were found in those made from different grape varieties, of which phenols were identified as major contributors to human health (Majkic, Torovic, Lesjak, Cetojevic-Simin, & Beara, 2019). Studies have further shown that drinking wine moderately helped to reduce the incidence of coronary heart diseases, and this was closely related to the composition and content of polyphenolics in wine, especially those with high levels of catechins, tannins and resveratrol (Shahidi & Ambigaipalan, 2015). In addition, such polyphenolics are also related to their antioxidant activity due to their ability to scavenge various free radicals (Ladnrault, Poucheret, Ravel, Gasc, Cros, & Teissedre, 2001). Hence, increasing attention is now being paid to improving phenolic substances by mixed fermentation. In this context, Liao et al. (Liao et al., 2023) used *L. fermentum*, *L. plantarum* and *S. thermophilus* for co-fermentation, with the results showing that the total phenolic, ferulic acid, rutin and quercetin-3-rhamnoside content was the highest when *L. fermentum* and *L. plantarum* were mixed in a ratio of 0.5:0.5. Thus, the current authors considered that the focus should be on polyphenolics in mixed fermented wines.

In this study, *Pichia kudriavzevii* was isolated from the wine grape production area of Shihezi, Xinjiang, China. To evaluate the strain’s performance in improving the antioxidant activity and aroma of wine, this work also explored mixed fermentation of Cabernet Sauvignon wine by *Pichia kudriavzevii* and *Saccharomyces cerevisiae*. Finally, with single fermentation by *Saccharomyces cerevisiae* acting as the control, the effects of *Pichia kudriavzevii* and *Saccharomyces cerevisiae* mixed fermentation on wine quality in terms of polyphenolics content, antioxidant activity, volatile aromatic compounds and aroma characteristics were investigated.

## Materials and methods

### Yeast strains and raw material

The *Pichia* (*P.*) *kudriavzevii* CCTCC 2017759 (China Type Culture Collection) and commercial *Saccharomyces* (*S.*) *cerevisiae* 7VA (EnotechUPM, Madrid, Spain) 7VA) were used to ferment wine through the co-inoculated and sequentially inoculated method. Both of them were maintained on YPD mediums (10 g/L yeast extract, 20 g/L peptone, 20 g/L glucose, 20 g/L agar).

Cabernet Sauvignon grape was collected from Shihezi City, Xinjiang Uygur Autonomous Region. The grapes were transported back to the laboratory under refrigerated conditions (4 °C) within 12 h after picking, and the subsequent treatment was completed within 24 h.

### Winemaking process

Micro-vinification of wine was carried out in a laboratory-based setting. In brief, after crushing, Cabernet Sauvignon grapes were added with 50 mg/L SO_2_, and mixed completely to avoid oxidation, followed by cold dipping at 4 °C for 24 h. Broken grapes were placed into 30 L glass bottles, and inoculated with yeast to start fermentation. The fermentation temperature was controlled at 25–27 °C, and the cap pressing treatment was carried out every 24 h to fully contact the grape peel and grape juice. Finally, adding 50 mg/L SO_2_ to stop fermentation when reducing sugar is below 4 g/L.

Yeast inoculation methods: co-inoculation: *P. kudriavzevii* M759 and *S. cerevisiae* 7VA were inoculated simultaneously in different ratios, and the *P. kudriavzevii* M759/*S. cerevisiae* 7VA ratios were 1:1 (C1:1), 1:5 (C1:5), 1:10 (C1:10). Sequential inoculation: *S. cerevisiae* 7VA was inoculated 48 h after inoculation with *P. kudriavzevii* M759, and the *P. kudriavzevii* M759/ *S. cerevisiae* 7VA ratios were 1:1 (S1:1), 1:5 (S1:5), 1:10 (S1:10). The number 1 in previous ratios represented 10^6^ CFU/mL. All experiments were repeated in triplicates.

### Determination of total phenolic content

The determination of total phenolic content was performed according to Folin–Ciocalteu colorimetric method (Wu et al., 2015). Dilute wine properly with distilled water. Added 1.5 mL Folin-Ciocalteu reagent (0.2 mol/L) to 0.3 mL diluted wine, and adjusted the absorbance in the range of 0.20–0.80. After the reaction was carried out for 5 min, 1.2 mL Na_2_CO_3_ (0.7 mol/L) was added. The sample reacted for 120 min at room temperature and in the dark, the absorbance was measured at 760 nm. All experiments were repeated in triplicates. A standard solution of 0–1000 μg/mL gallic acid was prepared, and a standard curve was prepared for calculating the total phenolic content in wine.

### Determination of total flavonoid content

The determination of total flavonoid content refers to the method described by Cao et al. (Cao, Yang, & Zheng, 2013). One mL wine sample was placed in test tube, 0.1 mL distilled water and 0.075 mL 5% (w/w) NaNO_2_ solution were added, and kept at room temperature for 5 min. Then 0.15 mL 10% (w/w) AlCl_3_·6H_2_O solution was added, and 0.1 mL 1 mol·L^-1^ NaOH solution was added after 5 min. After mixing, the absorbance was measured at 510 nm. All experiments were repeated in triplicates. A standard solution of 0–100 μg/mL rutin was prepared, and a standard curve was prepared for calculating the total flavonoid content in wine.

### Determination of phenolic compounds

Phenolic compounds were analyzed by HPLC at 280 nm using an Agilent 1100 system (Agilent Technologies, Palo Alto, CA, USA) equipped with an Agilent 1100 series VWD and a Luna C18 column (250 × 4.6 mm, 5 μm particle size; Phenomenex, Macclesfield, UK). The mobile phase was comprised of 2% (v/v) acetic acid (A), and acetonitrile (B) at flow rate of 1.0 mL/min. The gradient program was from 15% B for 15 min, from 15% to 40% B for 25 min, from 40% to 30% B for 5 min, from 30% to 20% B for 5 min, from 20% to 15% B for 5 min and 15% B for 10 min. The injection volume was 10 μL. The column temperature was 30 ℃. The detection wavelength was 280 nm. All wine samples were filtered through 0.22 μm nylon filter before HPLC analysis. All experiments were repeated in triplicates.

### Antioxidant capacity

The determination of total antioxidant capacity refers to the method described by Shopska et al. (Shopska, Kostova, Zarcheva, Teneva, Denev, & Kostov, 2021). Two hundred and fifty µL of wine was added to 2.25 mL of 0.06 mmol/L DPPH solution in methanol; the mixture was allowed to react for 15 min in the dark, then the absorbance at 517 nm was determined. Samples prepared with methanol as a control. The antioxidant activity was determined by a standard curve using Trolox as standard, and the results were expressed as µM Trolox equivalents per 1 mL for wine:

$$\mathrm{DPPH}\%=\frac{\left(A_{517}^{k}-A_{517}\right)}{A_{517}^{k}}\times 100,\;\%\;inhabition$$

$$\mathrm{DPPH}=K_{p}\times \frac{\mathrm{DPPH}\%+0.6711}{0.341}\times 10^{-3},\;\mu mol\;TROLOX/mL$$

where: Kp-dilution coefficient of the wine.

7 × 10^-3^ mol/L ABTS solution and 2.45 × 10^-3^ mol/L potassium persulfate solution were mixed in the ratio of 1: 1, and the mixture was placed in the dark for 16 h. The mixture was diluted with methanol until the absorbance was 1.1 ± 0.1. The absorbance was measured against methanol at 734 nm. The wine was diluted with distilled water to achieve A734 in the range of 0.2–0.9. Methanol as blank sample, 0.15 mL MeOH + 2.85 mL ABTS and 0.15 mL diluted wine and 2.85 mL ABTS as control sample. The measurement was carried out at 735 nm, and the results were calculated according to the following formula:

$$I=100\times \frac{A_{1}-A_{2}}{A_{1}},\;\%$$

where: A_1_—the absorbance of the control against the blank sample; A2—the absorbance of the working sample against the blank sample;

The concentration of Trolox solution was determined by standard curve. The antiradical activity was determined after 120 min, expressed in mM Trolox for wine (mM TE/mL):

$$C_{\mathrm{Trolox}}=\frac{I+1.6762}{0.1164},\;\mu mol\;TE$$

$$\mathrm{AO}A_{\mathrm{ABTS}}=K_{p}\times C_{\mathrm{Trolox}}\times 10^{-3},\;\mu mol\;TE/mL$$

$$K_{p}=K_{\mathrm{MeOH}}\times K_{\mathrm{water}}$$

where: Kp-dilution coefficient of the wine.

Preparation of 300 mM acetate buffer solution with pH 3.6, a stock solution, by using 3.1 g of C_2_H_3_NaO_2_·3H_2_O and 16 mL of C_2_H_4_O_2_. 10 mM TPTZ solution prepared in 40 mM HCl and 20 mM FeCl_3_·6H_2_O solution. The working solution is a mixture of TPTZ solution, FeCl_3_·6H_2_O solution and acetate buffer in a ratio of 1:1:10. 150 μL of wine reacted with 2850 μL of FRAP solution for 4 min in the dark. The absorbance of the wine was measured at 593 nm against a blank prepared with methanol. A standard curve using Trolox as a standard was used to determine the antioxidant activity of wine, and the results were expressed as μmol/L Trolox equivalents per 1 mL of wine:

$$\mathrm{FRAP}=\frac{A_{593}+0.0235}{0.0024}K_{p}\times 10^{-3},\;\mu mol\;TROLOX/mL$$

where: Kp-dilution coefficient of the wine.

Preparation of 0.01 mol/L solution of CuCl_2_·2H_2_O, 7.5 × 10^−3^ mol/L solution of neocuproin and acetate buffer with pH = 7 for subsequent use. If necessary, the wine was diluted to a suitable dilution with methanol. 1 mL of CuCl_2_·2H_2_O and 1 mL of acetate buffer and 1 mL of neocuproin and 0.5 mL of extract and 0.6 mL of distilled water as a working sample; 1 mL of CuCl_2_·2H_2_O and 1 mL of acetate buffer and 1 mL of neocuproin and 1.1 mL of distilled water as a blank sample. All samples were homogenized to assay and allowed to stand at room temperature for 30 min. the absorbance of wine against the blank sample at 517 nm was determined, and the results were calculated according to the following formula:

$$\mathrm{AOA}=\frac{A_{450}-0.0161}{0.0018}K_{p}\times 10^{-3},\;\mu mol\;Trolox/mL$$

where: Kp-dilution coefficient of the wine.

### Qualitative and quantitative analysis of the aroma compounds

Headspace solid-phase microextraction (HS-SPME) was carried out following the methodology of Wang et al. (Wang, Li, Dizy, Ullah, Sun, & Tao, 2017) with some modifications. In a nutshell, a 15 mL glass phial with 8 mL wine sample, 1 g sodium chloride, 5 μL of 1000 mg/L 2-octanol (≥99%, PolyScience Co., Niles, IL, USA) as internal standard substance and a magnetic stirring bar were added, sealed with the headspace sealing bottle cap. The vial containing the sample was placed on a magnetic stirrer and balanced at 40 ℃ for 15 min at 150 rpm. Then, the SPME fiber was exposed to the headspace of the sample bottle and extracted for 15 min (40 ℃, 150 rpm) (Wang et al., 2017).

Analysis of aroma compounds by GC–MS. The GC–MS system, comprised a TRACE 1310 gas-chromatograph and an ISQ LT Single Quadrupole mass spectrograph (Thermo SCIENTIFIC, USA), was used to isolated and identified the aroma compounds. A DB-WAX capillary column (60 m × 0.25 mm × 0.25 μm, Agilent J&W, USA) was used. The aroma compounds were desorbed at 250 ℃ for 8 min in splitless mode, carrier gas was ultrapure helium (99.999%) at a flow rate of 1 mL/min. GC program started at 40 ℃, then from 40 ℃ to 130 ℃ at a rate of 3 ℃/min, and then ramped from 130 ℃ to 250 ℃ at a rate of 5 ℃/min, 10 min at 250 ℃. The temperature of the injector was set at 250 ℃, as were the transfer line and ion source. The spectra were obtained on electron ionization (EI) at 70 eV with a scan range of 25–450 amu and an interval of 0.2 s.

Qualitative and quantitative analysis of all the aroma compounds were identified by matching retention index (RI) and the NIST MS library. The relative volatile abundance of each compound was calculated using the following formula: peak area/area of internal standard × internal standard concentration.

The odor activity value (OAV) was calculated using the equation OAV = c/OT, where c is the content of the compound in the Cabernet Sauvignon wine and OT is the odor threshold value. The compounds with OAV > 1 are generally considered to be odor-active compounds (Tunick, 2014).

### Statistical analysis

All experimental data were mean values of three parallel experiments and were expressed as mean ± standard deviation. A significant difference between the groups was assessed using Duncan’s test at a 5% level (*P* < 0.05) by SPSS 20 (IBM, Chicago, USA). The principal component analysis (PCA) was used to correlate the relative abundance (μg/L) of each volatile compound using the SIMCA 14.1 software (Biometric Software Developer Umetrics, Umeå, Sweden). The R version 4.2.0 (Vigorous Calisthenics) was used to generate a heatmap to analyze the effect of different fermentation methods on wine flavor.

## Results and discussion

### Analysis of physicochemical properties

The physical and chemical indexes of seven wine samples were detected, of which five were significantly different from each other (*P* < 0.05). As shown in Table S1, the residual sugar content in the wines was lower than 4 g/L, indicating that mixed fermentation with *P. kudriavzevii* M759 and *S. cerevisiae* 7VA ensured the smooth progress of wine alcohol fermentation. Fig. S1 further shows that the content of titratable acid in mixed fermentation wines (S1:1, S1:5, S1:10 and C1:10) was significantly lower than that in CK, which was consistent with the research results of Padilla et al. (2016). However, it is worth noting that C1:1 and C1:5 was not significantly different from CK in terms of the titratable acid, and this could have been due to the effects of simultaneous inoculation with *S. cerevisiae* 7VA on *P. kudriavzevii* M759. The pH was also closely related to the content of titratable acid. While no obvious changes were noted in the total phenolic content of the wines, the total amount of anthocyanin in mixed fermented ones was higher than that of CK, with this change contributing to improved color stability of the wine. This difference in total anthocyanin content could, in fact, be attributed to the longer duration of the mixed fermentation (14 d) compared to CK (10 d), as this prolongs the time for anthocyanins combining (Loira, Morata, Comuzzo, Callejo, & Suárez-Lepe, 2015).

### Quantitative assessment of phenolic compounds

Grapes are rich sources of phenolic compounds, which have protective effects on human health, including antioxidant, anti-inflammatory and heart protection. In addition, these phenolic compounds also play an important role in the visual and taste quality of red wine, while contributing to the latter’s complexity and stability (Minnaa, Plessi, Paulsen, Ntushelo, Jolly, & Toit, 2017). In order to determine the effects of *P. kudriavzevii* M759 on the composition and content of phenolic compounds in wine, nine different polyphenols, including ferulic acid, vanillic acid, chlorogenic acid, rutin, quercetin, kaempferol, (-)-catechin, (-)-epicatechin and procyanidin B1, were quantitatively analyzed, with the results shown in Table S2.

**Fig. 1:**
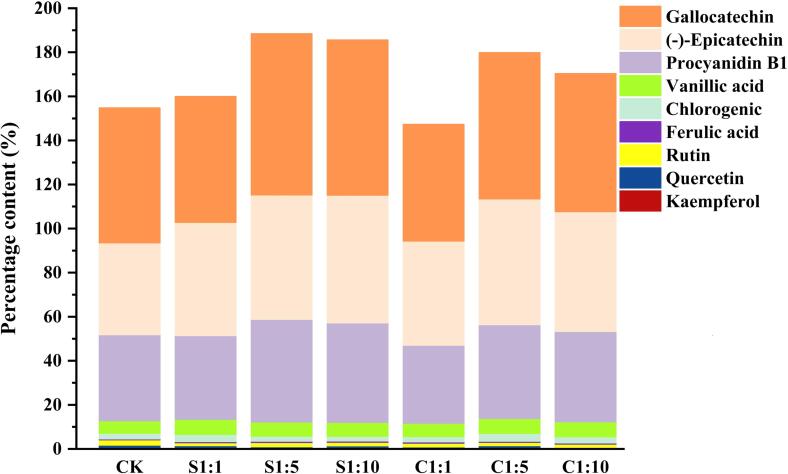
Percentage content of phenolic compounds in wine with 6 different fermentation methods. CK represents *S. cerevisiae* 7VA single bacteria fermented wine. C1:1, C1:5, C1:10 represent co-inoculation of *S. cerevisiae* 7VA and *P. kudriavzevii* M759 at a ratio of 1:1, 1:5, 1:10, respectively. S1:1, S1:5, S1:10 represent sequential inoculation of *S. cerevisiae* 7VA and *P. kudriavzevii* M759 at a ratio of 1:1, 1:5, 1:10, respectively.

As shown in Fig. 1, flavan-3-ols ((-)-catechin, (-)-epicatechin and procyanidin B1), accounting for >91% of the total monophenols, were the main phenolic substances identified in wine in this study. Mixed fermentation using *P. kudriavzevii* M759 and *S. cerevisiae* 7VA actually influences the concentration of flavan-3-ols in wine. When the inoculation ratio of *P. kudriavzevii* M759 and *S. cerevisiae* 7VA reached 1:5 and 1:10, respectively, the content of nine phenolic compounds in the mixed fermented wine was significantly higher than that in CK (*P* > 0.05). In addition, the contents of S1:5 and S1:10 were the highest, with increases of 21.76% and 19.92% respectively compared with CK. Generally, β-glucosidase activity of *S. cerevisiae* 7VA is lower than that of non-*Saccharomyces cerevisiae*, and extracellular β-glucosidase activity of *Pichia* was abnormally high (Caridi, Cufari, Locino, Palumbo, & Tedesco, 2004). Therefore, the increase in phenolic content during mixed fermentation with a higher inoculation ratio of *P. kudriavzevii* M759 could be due to the latter’s higher enzymatic activity as β-glucosidase hydrolyzes the glycoside forms of some phenolic compounds in wine (Villena, Iranz, Otero, & Perez, 2010). In consistent with the results of previous study, phenols attach to yeast cell walls and are lost as the yeast flocculates or dies, which may be the reason why the content of flavan-3-ol in C1:1 was lower than that in CK (Zhang, Zhang, Sirisena, Gan, & Fang, 2021). Strong interactions between *P. kudriavzevii* M759 and *S. cerevisiae* 7VA could also have led to the loss of phenolic compounds when the inoculation ratio is low. However, from the results presented in Table S2, for single phenolic substances, S1:5 had the highest (-)-catechin and procyanidin B1 content (73.23 mg/L and 46.59 mg/L), while S1:10 had the highest amount of (-)-epicatechin (57.95 mg/L). It is worth noting that compared with CK, mixed fermentation reduced the flavonoids content (quercetin, rutin and kaempferol) in wine. Similar to the report (Markkinen, Laaksonen, Nahku, Kuldjärv, & Yang, 2019), *Lactobacillus plantarum* DSM 10492 and *Lactobacillus plantarum* DSM 100813 fermented reducing the total flavonol glycoside content of chokeberry juices. At the same time, there were also changes in the phenolic acids under different fermentation modes, but only slight ones within a limited range. The above data are consistent with the data presented in Table S2 and Fig. 1, demonstrating that high inoculation ratio of *P. kudriavzevii* M759 (S1:5 and S1:10) can affect the microbial metabolism of phenolics and potentially enhance phenolic contents compared with single fermentation of *S. cerevisiae* 7VA fermentation.

### Effect of mixed fermentation on antioxidant activity of wine

Grape is rich in polyphenols, which have strong antioxidant activities. In this study, the effects of *P. kudriavzevii* M759 on the antioxidant activity of wines produced by different fermentation methods were investigated using four in vitro tests, with the results were shown in Table 1.

**Table 1:** Effects of different fermentation methods on antioxidant activity of the Cabernet Sauvignon wine samples.

Antioxidant activity	Samples						
	CK	C1:1	C1:5	C1:10	S1:1	S1:5	S1:10
DPPH	5.58 ± 0.22^d^	5.61 ± 0.25^d^	5.74 ± 0.27^c^	5.75 ± 0.21^c^	5.6 ± 0.19^d^	6.59 ± 0.34^a^	6.14 ± 0.21^b^
ABTS	10.96 ± 0.32^c^	9.59 ± 0.29^f^	10.21 ± 0.23^e^	11.06 ± 0.25^c^	10.71 ± 0.22^d^	12 ± 0.34^b^	12.48 ± 0.22^a^
FRAP	9.63 ± 0.55^d^	9.32 ± 0.26^e^	11.29 ± 0.54^c^	11.53 ± 0.42^c^	8.61 ± 0.4^f^	12 ± 0.35^b^	12.66 ± 0.37^a^
CUPRAC	17.57 ± 0.34^d^	16.46 ± 0.3^e^	18.58 ± 0.29^c^	19.26 ± 0.34^b^	17.56 ± 0.31^d^	18.57 ± 0.36^c^	22.03 ± 0.82^a^

Data are expressed as the mean ± standard deviation from replicate analyses (n = 3) of three replicate samples. The different lowercase letters in each row indicate significant differences between samples (*P* < 0.05).

The free radical scavenging ability (DPPH, ABTS) and reducing power (FRAP, CUPRAC) of wines produced by different fermentation methods were studied. The results indicated that all wine samples had antioxidant activity, with the Fig. S2 showing that most of the mixed fermentation methods improved the antioxidant. In particular, S1:10 showed the strongest FRAP, CUPRAC and ABTS (31.46 %, 25.38 % and 13.87 % higher than that of CK on average), while S1:5 had the strongest DPPH (18.1 % higher than that of CK), and these reflected the trend in phenolics content. Hence, it was speculated that the accumulation of phenolics during fermentation was the key reason for the enhanced antioxidant capacity (Wang et al., 2021). As reported by Li et al. (Li et al., 2021), the *P. kudriavzevii* M759 used in this study could also improve the antioxidant activity of wine. It is worth noting that under the same inoculation ratio, the antioxidant activity of sequential inoculation was higher than that of co-inoculation, which was closely related to the interaction between *P. kudriavzevii* M759 and *S. cerevisiae* 7VA. Compared with CK, all four measured antioxidant activities were lower after mixed fermentation (S1:1 and C1:1) with a low inoculation ratio of *P. kudriavzevii* M759. This could be due to the fact that, at lower inoculation amounts, *P. kudriavzevii* M759 was not be the dominant strain during the early stages of fermentation.

### Correlation assay between antioxidant activity and phenolic compounds

**Fig. 2:**
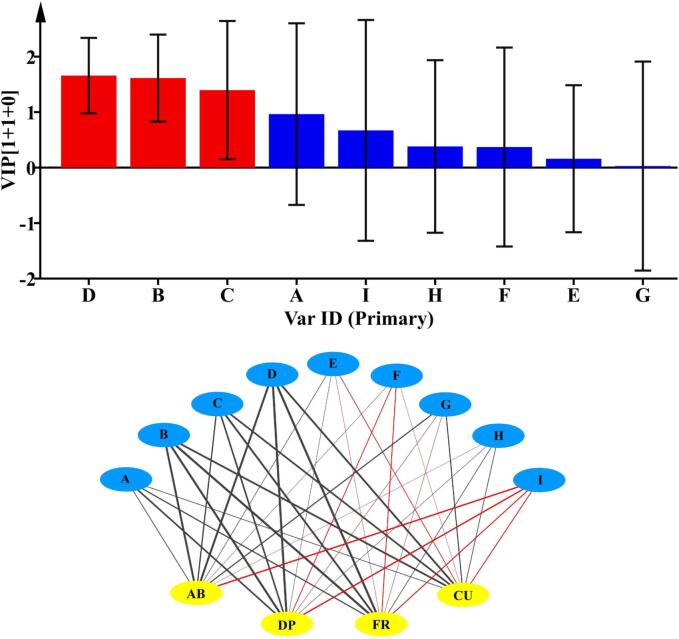
Correlation analysis of phenolic compounds and antioxidant capacity. A, B, C, D, E, F, G, H and I represent ferulic acid, (-)-catechin, (-)-epicatechin, procyanidin B1, rutin, quercetin, kaempferol, vanillic acid, chlorogenic acid, respectively. AB, DP, FR and CU represent the 4 antioxidant indexes ABTS, DPPH, FRAP, CUPRAC, respectively.

In order to further study the effects of *P. kudriavzevii* M759 on the antioxidant capacity of wine, the correlation between phenolic concentration and antioxidant capacity was analyzed based on O2PLS. The results showed that the antioxidant capacity of wine was closely related to the flavan-3-ols content, with the correlation between different polyphenols and antioxidant capacity being also significantly different. Therefore, it is likely that different polyphenols contributed differently to the antioxidant capacity of wine. In particular, the VIP values of (-)-catechin, (-)-epicatechin and procyanidin B1 were >1, indicating that these substances were the main contributors to the antioxidant capacity of wine. Fig. 2 showed that ferulic acid was positively correlated with four antioxidant indexes. (-)-Catechin, (-)-epicatechin and procyanidin B1 were strongly positively correlated with the four antioxidant indexes. In addition, there was a positive correlation between rutin, DPPH and ABTS, between quercetin and ABTS, between kaempferol, ABTS and CUPRAC, as well as between vanillic acid, DPPH, CUPRAC and FRAP. However, chlorogenic acid did not contribute to the antioxidant capacity of wine.

### Aroma profiles of wines

The volatile compounds in different wines were also characterized to study the effects of *P. kudriavzevii* M759 and different inoculation methods on the aroma of Cabernet Sauvignon wine. As shown in Table S3, a total of 47 volatiles from 6 different categories were identified for seven different inoculation methods, and these included 20 esters, 12 alcohols, 7 acids, 4 phenylethyls, 3 terpenoids, and 1 other compound. Moreover, 14 compounds with OAV > 0.1, including 7 esters, 1 alcohol, 3 acids and 3 phenylethyls. The further results showed that there were significant differences in total volatile compounds content between pure and co-fermentation of *S. cerevisiae* 7VA, and the content of volatile compounds in individual samples was related to yeast species and inoculation methods involved in fermentation. Eventually, the interactions were found that interactions between *P. kudriavzevii* M759 and *S. cerevisiae* 7VA had important effects on the aroma of Cabernet Sauvignon wine.

Higher alcohols are important aroma compounds in wine, and they are mainly produced by live yeast cells through amino acid deamination during fermentation (Hazelwood, Daran, Maris, Pronk, & Dickinson, 2008). According to Table 2, only the OAV of isoamyl alcohol is >1 in all Cabernet Sauvignon wine samples, which helped to produce green and ethereal-fruit notes (Viana, José, Salvador, & Manzanares, 2009). When compared with single fermentation of *S. cerevisiae* 7VA and mixed fermentation, the content of isoamyl alcohol in mixed fermentation wine was higher than that in single fermentation except C1:1, and the content of isoamyl alcohol in sequential inoculation wine was higher than that in common inoculation under the same inoculation ratio. It is worth noting that under the same inoculation method, the content of isoamyl alcohol increased with the increase of inoculation ratio of *P. kudriavzevii* M759. Some higher alcohols, such as isobutanol and hexanol, which have special aroma may also help to improve the aroma characteristics of wine. However, the mixed fermentation approach did not significantly increase the content of these substances, with the hexanol content even decreasing, especially after co-inoculation. These results were similar to those reported by Loira et al. (Loira et al., 2015).

**Table 2:** Volatile compounds with OAV > 0.1 in Cabernet Sauvignon wine samples.

Compound	Threshold (μg/L)	OAV						
		CK	C1:1	C1:5	C1:10	S1:1	S1:5	S1:10
**Esters**								
Ethyl acetate	5	>1	>1	>1	>1	>1	>1	>1
Ethyl butyrate	0.18	>1	>1	>1	>1	>1	>1	>1
Isoamyl acetate	30	>1	>1	>1	>1	>1	>1	>1
Ethyl hexanoate	2.2	>1	>1	>1	>1	>1	>1	>1
Ethyl heptanoate	2	>1	>1	>1	>1	>1	>1	>1
Ethyl octanoate	15	>1	>1	>1	>1	>1	>1	>1
Ethyl decanoate	23	>1	>1	>1	>1	>1	>1	>1
**Alcohols**								
Isoamyl alcohol	30,000	>1	>1	>1	>1	>1	>1	>1
A**cids**								
Hexanoic acid	420	>1	>1	>1	>1	>1	>1	>1
Octanoic acid	500	>1	>1	>1	>1	>1	>1	>1
Decanoic acid	1000	>0.1	>0.1	>0.1	>1	>0.1	>0.1	>0.1
**Phenylethyls**								
Phenethyl acetate	250	>0.1	>0.1	>1	>1	>1	>1	>1
Benzyl alcohol	2000	>0.1	>0.1	>0.1	>0.1	>0.1	>0.1	>0.1
Phenylethanol	14,000	>1	>1	>1	>1	>1	>1	>1

**Fig. 3:**
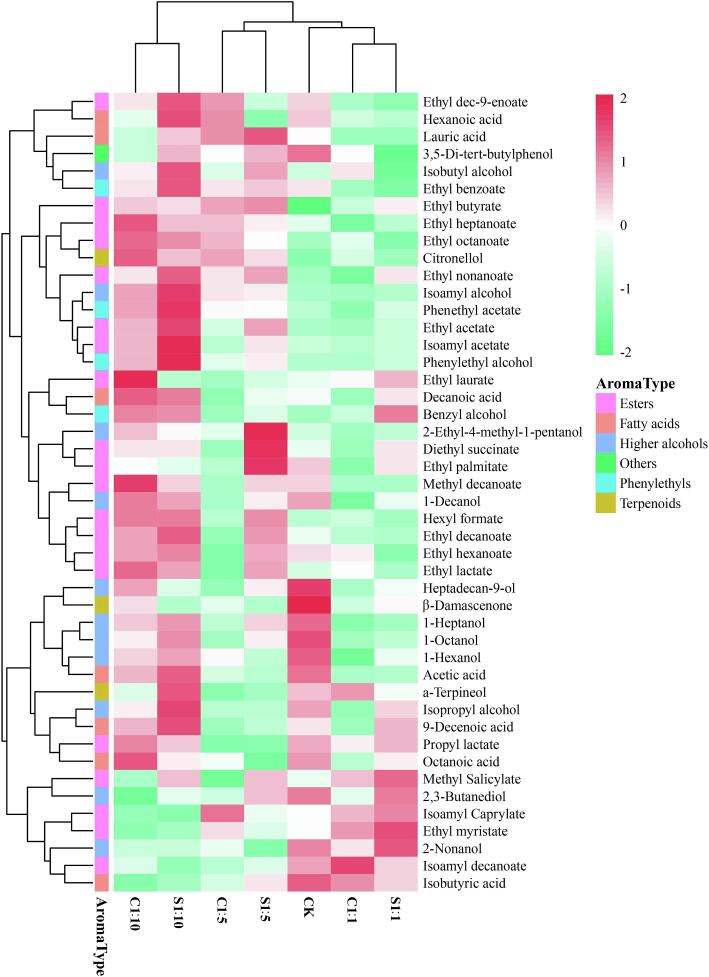
Heatmap cluster analysis of seven samples of the wine. CK represents *S. cerevisiae* 7VA single bacteria fermented wine. C1:1, C1:5, C1:10 represent co-inoculation of *S. cerevisiae* 7VA and *P. kudriavzevii* M759 at a ratio of 1:1, 1:5, 1:10, respectively. S1:1, S1:5, S1:10 represent sequential inoculation of *S. cerevisiae* 7VA and *P. kudriavzevii* M759 at a ratio of 1:1, 1:5, 1:10, respectively.

Esters are considered to be important aroma compounds in wine as they can give wine floral and fruity flavors (Swiegers, Bartowsky, Henschke, & Pretorius, 2005). Table S3 shows the 20 esters, mainly acetate esters and ethyl esters, that were identified in Cabernet Sauvignon wine. It is noteworthy, 6 of the 7 esters with OAV > 1 were ethyl esters. Acetate esters are formed by the catalysis of higher alcohols and acetyl-coA by alcohol acyl-transferases (AAT) in yeast cells (Peddie, 1990). Fig. 3 further shows that a higher acetate content in wine by mixed fermentation with high inoculation ratio *P. kudriavzevii* M759 was higher than that of single fermentation of *S. cerevisiae* 7VA, and the sequential inoculation was higher than co-inoculation. These results could be related to the expression level encoded by IAH esterase in *P. kudriavzevii* M759 as well as the sequential inoculation method which could have been more conducive to interactions between *P. kudriavzevii* M759 and *S. cerevisiae* 7VA. Ethyl esters are formed by an enzyme-catalyzed condensation reaction between ethanol and an acyl-CoA component. Fig. 3 showed that most of the low-grade fatty acid ethyl ester content in the co-fermentation wine was higher compared with the single fermentation based on *S. cerevisiae* 7VA, with the amounts of ethyl acetate (7262 μg/L), ethyl octanoate (2105 μg/L) and propyl lactate (6079 μg/L) being particularly significant. The content of other ethyl esters also increased, but only within a limited range.

Table S3 showed that all Cabernet Sauvignon wines contained seven types of fatty acids, with fermentation performed using only *S. cerevisiae* 7VA having the highest total fatty acid content. Hexanoic acid, octanoic acid, decanoic acid and 9-deenoic acid belonged to the group of medium chain fatty acids, while the content of single fatty acids in wine, fermented with a high inoculation ratio of *P. kudriavzevii* M759 was higher than that fermented with only *S. cerevisiae* 7VA. In fact, the higher content could produce cheese flavor similar to butter and increase the complexity of wine aroma (Francis & Newton, 2005). However, when the fatty acid content is too high, it can produce an irritant odor which has a negative impact on the flavor of wine (Ribéreau-Gayon, Dubourdieu, Donèche, & Lonvaud, 2006). In the samples under study, octanoic acid content was the highest, with a concentration range of 4593–5752 μg/L, and it was followed by hexanoic acid for which the concentration range was 1394–1586 μg/L. These concentrations were much lower than the levels at which they become unpleasant. At the same time, lower levels of volatile fatty acids can effectively prevent ester hydrolysis and contribute to a balance in the wine’s aroma.

Phenylethyls and terpenes have significant effects on the fruit and flower aroma of wine, thus helping to improve the latter’s quality (Viana et al., 2009). In particular, 2-phenylethanol is an interesting substance in wine. It is mainly synthesized by the Ehrlich pathway and l-phenylalanine ammonia transfer, and it can bring rose-like fragrance to wines while giving a honey flavor (Swiegers, Bartowsky, Henschke, & Pretorius, 2005). In this study, 2-phenylethanol accounted for 97.04%–98.08% of the total content of phenylethyls. Although the sensory threshold of 2-phenylethanol was high (10000 μg/L), their concentrations were much higher than their sensory threshold. Moreover, the phenylethanol content in the mixed fermentation wine (C1:5, C1:10, S1:5, S1:10), produced with a high inoculation ratio was significantly higher than that produced by single fermentation of *S. cerevisiae* 7VA. The highest content in S1:10 was 117848 μg/L, which was 1.96 times that of CK, indicating that co-fermentation could bring more intense rose-like aroma to wine. Although 0.1 < OAV < 1 for phenethyl acetate and benzyl alcohol, they may have synergistic effects on aroma (Wang et al., 2017). Terpenes are usually combined with glycosides to yield non-volatile compounds during wine fermentation. In this context, β-glucosidase is the key enzyme that hydrolyzes the odorless grape glycosidic precursors to release volatile compounds, such as phenylethanol and citronellol in this study, with co-fermentation increasing the amounts of such compounds (Waterhouse, Sacks, & Jeffery, 2016). Therefore, it was speculated that *P. kudriavzevii* M759 had strong β-glucosidase activity during wine-making. It can be seen from Fig. 3 that, unlike the results reported by Rodrıguez-Bencomo et al. (Rodrıguez-Bencomo, Méndez-Siverio, Pérez-Trujillo, & Cacho, 2008), using *P. kudriavzevii* M759 and *S. cerevisiae* 7VA did not increase the terpenes content. Instead, the amount of β-damascenone in the mixed fermentation wine was lower compared with fermentation using only *S. cerevisiae* 7VA. At the same time, the sensory threshold of this substance was extremely low. Hence, even though the content was reduced, it also contributed to the flavor of the wine.

**Fig. 4:**
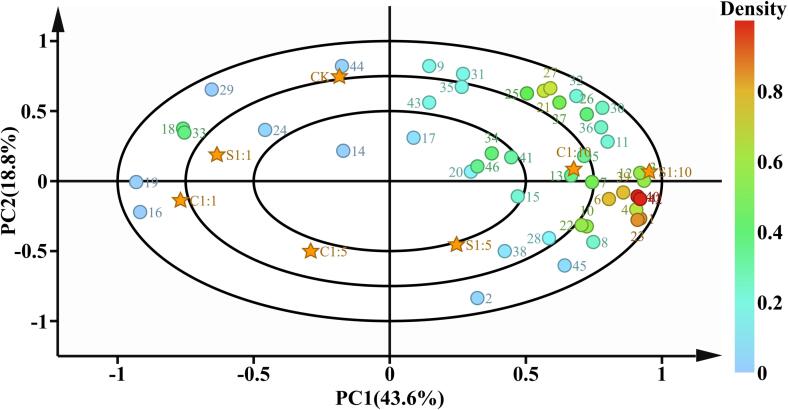
Principal component analysis (PCA) of volatile compound formation. CK represents *S. cerevisiae* 7VA single bacteria fermented wine. C1:1, C1:5, C1:10 represent co-inoculation of *S. cerevisiae* 7VA and *P. kudriavzevii* M759 at a ratio of 1:1, 1:5, 1:10, respectively. S1:1, S1:5, S1:10 represent sequential inoculation of *S. cerevisiae* 7VA and *P. kudriavzevii* M759 at a ratio of 1:1, 1:5, 1:10, respectively.

In order to further study the effects of *P. kudriavzevii* M759 and different mixed fermentation methods on the volatile compounds in Cabernet Sauvignon wine, 46 volatile compounds were analyzed by principal component analysis. PC1 and PC2 were 43.6% and 18.8%, respectively, explaining most of the variations. The load diagram of volatile compounds and the distribution of wine samples were as shown in Fig. 4. Different starting strains, inoculation methods and inoculation ratios of *S. cerevisiae* 7VA to *P. kudriavzevii* M759 had obvious effects on the aroma of wine. In the first quadrant, there were a variety of volatile aroma compounds near S1:10 and C1:10, including esters and phenylethyls, but wine samples in other quadrants were far away from these volatile compounds. This indicated that the mixed fermentation mode with higher inoculation rate of *P. kudriavzevii* M759 had more esters and phenylethys, which improved the aroma characteristics of Cabernet Sauvignon wine.

## Conclusion

This study showed the contribution of *P. kudriavzevii* M759 to phenolics, antioxidant capacity and flavor of Cabernet Sauvignon wines. The results showed that the mixed fermentation of *P. kudriavzevii* M759 and *S. cerevisiae* 7VA can ensure the smooth progress of wine alcoholic fermentation, and helps to improve the color stability of wine. Fermentation methods had a significant effect on phenolic compounds in wine, and *P. kudriavzevii* M759 helped to increase the content of monomeric phenols in wine, with the highest content in S1:5 and S1:10. Compared with CK, mixed fermentation enhanced the antioxidant activity of wine, and the antioxidant activity of S1:10 was the strongest. The analysis of volatile components showed that the mixed fermentation of *P. kudriavzevii* M759 and *S. cerevisiae* 7VA increased the alcohols, esters and phenylethyls in the wine. The sequential inoculation of *P. kudriavzevii* M759 with a higher inoculation ratio such as S1:10 was more conducive to the formation of volatile aroma compounds in wine.

## Ethical approval

This article does not contain any studies with animals performed by any of the authors.

## CRediT authorship contribution statement

**Wenhan Liu:** Data curation, Writing – original draft. **Rujie Ji:** . **Ayinuer Aimaier:** . **Jinkui Sun:** Writing – review & editing. **Xilei Pu:** Writing – review & editing. **Xuewei Shi:** Funding acquisition, Resources. **Weidong Cheng:** . **Bin Wang:** .

## Declaration of Competing Interest

The authors declare that they have no known competing financial interests or personal relationships that could have appeared to influence the work reported in this paper.

## Data Availability

No data was used for the research described in the article.
