# Supplementary material for: Adjustment of impact phenolic compounds, antioxidant activity and aroma profile in Cabernet Sauvignon wine by mixed fermentation of Pichia kudriavzevii and Saccharomyces cerevisiae
Source: Food Chem X. 2023 Apr 19;18:100685. doi: 10.1016/j.fochx.2023.100685 (PMC10149247; doi:10.1016/j.fochx.2023.100685)
Supplement: Supplementary data 1 [file mmc1.docx]

**Supplementary Information for**

**Adjustment of impact phenolic compounds, antioxidant activity and aroma profile in Cabernet Sauvignon wine by mixed fermentation of *Pichia kudriavzevii* and *Saccharomyces cerevisiae***

Wenhan Liu^a^, Rujie Ji^a^, Ayinuer Aimaier^a^, Jinkui Sun^a^, Xilei Pu^a^, Xuewei Shi^a^, Weidong Cheng^a, *^, Bin Wang^a, *^

^a^ *College of Food Science and Technology, Shihezi University, Shihezi 832000, China*

* Correspondence: *E-mail addresses: W. C.: cwd0221@163.com; B. W.:* [*binwang0228@shzu.edu.cn*](mailto:binwang0228@shzu.edu.cn)

Tel.:86-0993-2058093

**Contents**

[Supplementary Tables 3](#_Toc85186929)

[Supplementary Table S1 Effects of different fermentation methods on physicochemical indexes of the Cabernet Sauvignon wine samples 3](#_Toc85186930)

[Supplementary Table S2 Content of the polyphenols in the Cabernet Sauvignon wine samples 4](#_Toc85186931)

[Supplementary Table S3 Content of the aroma components in the Cabernet Sauvignon wine samples 5](#_Toc85186930)

[Supplementary Figures 8](#_Toc85186929)

[Supplementary Fig. S1 Effects of different fermentation methods on physical and chemical indexes of wine. pH (black), residual sugar (red), titratable acid (blue) and total phenols (green), total anthocyanin (purple) 8](#_Toc85186930)

[Supplementary Fig. S2 Effects of different mixed fermentation methods on four antioxidant activities of wine 9](#_Toc85186931)

Supplementary Tables

Supplementary Table S1 Effects of different fermentation methods on physicochemical indexes of the Cabernet Sauvignon wine samples

| Samples | index | | | | |
| --- | --- | --- | --- | --- | --- |
|  | pH | Residual sugar  (mg/L) | Titratable acid  (mg/L) | Total phenols  (mg/L) | Total anthocyanin  (mg/L) |
| CK | 3.43±0.02^e^ | 3.69±0.13^a^ | 4.86±0.06^a^ | 1003.77±14.3^e^ | 444.97±20.79^e^ |
| C1:1 | 3.45±0.02^d^ | 3.49±0.18^bc^ | 4.81±0.06^b^ | 1083.17±13.38^d^ | 470.18±12.56^d^ |
| C1:5 | 3.47±0.03^d^ | 3.53±0.06^b^ | 4.79±0.06^b^ | 1083.33±17.06^d^ | 477.32±14.51^d^ |
| C1:10 | 3.57±0.01^c^ | 3.45±0.08^c^ | 4.54±0.07^c^ | 1084.33±34.19^d^ | 522.94±7.41^b^ |
| S1:1 | 3.58±0.03^c^ | 3.43±0.08^c^ | 4.56±0.1^c^ | 1129.6±36.32^b^ | 507.87±10.88^c^ |
| S1:5 | 3.63±0.01^b^ | 3.41±0.17^d^ | 4.4±0.07^d^ | 1145.43±24.5^a^ | 518.24±3.5^b^ |
| S1:10 | 3.72±0.02^a^ | 3.38±0.1^e^ | 4.22±0.09^e^ | 1113.1±26.69^c^ | 562.94±8.36^a^ |

Data are expressed as the mean ± standard deviation from replicate analyses (n=3) of three replicate samples. The different lowercase letters in each row indicate significant differences between samples (*P* < 0.05).

# **Supplementary Table S2** Content of the polyphenols in the Cabernet Sauvignon wine samples

| Polyphenol | Content (mg/L) | | | | | | |
| --- | --- | --- | --- | --- | --- | --- | --- |
|  | CK | C1:1 | C1:5 | C1:10 | S1:1 | S1:5 | S1:10 |
| (-)-Catechin | 61.24±2.05^c^ | 53.02±3.57^d^ | 73.23±1.35^a^ | 70.46±1.76^ab^ | 37.11±2.84^e^ | 66.44±1.36^b^ | 72.07±1.12^a^ |
| (-)-Epicatechin | 41.75±3.91^c^ | 37.28±1.82^d^ | 56.45±1.91^a^ | 47.95±2.36^b^ | 35.42±1.36^e^ | 57.06±2.58^a^ | 58.37±2.12^a^ |
| Procyanidin B1 | 39.03±1.63^b^ | 35.39±1.98^c^ | 46.59±1.06^a^ | 45.18±1.13^a^ | 28.01±1.49^d^ | 42.57±1.58^ab^ | 36±1.22^c^ |
| Rutin | 2.4±0.19^b^ | 2.72±0.19^a^ | 2.29±0.07^b^ | 1.62±0.15^d^ | 1.35±0.08^e^ | 2.15±0.21^c^ | 1.61±0.19^d^ |
| Quercetin | 1.63±0.08^a^ | 0.82±0.18^e^ | 0.92±0.09^d^ | 1.3±0.09^c^ | 1.41±0.08^b^ | 1.42±0.04^b^ | 0.52±0.08^f^ |
| Kaempferol | 0.08±0.01^b^ | 0.05±0.01^d^ | 0.04±0.01^e^ | 0.23±0.03^a^ | 0.06±0.01^c^ | 0.04±0.01^e^ | 0.03±0.01^f^ |
| Ferulic acid | 0.45±0.07^e^ | 0.61±0.08^a^ | 0.61±0.03^a^ | 0.58±0.09^b^ | 0.48±0.08^d^ | 0.49±0.11^d^ | 0.52±0.11^c^ |
| Vanillic acid | 5.68±0.19^f^ | 6.06±0.12^e^ | 6.41±0.15^c^ | 6.3±0.26^d^ | 6.82±0.25^a^ | 6.75±0.22^b^ | 6.75±0.12^b^ |
| Chlorogenic acid | 2.52±0.12^d^ | 2.37±0.14^e^ | 2.29±0.2^f^ | 2.13±0.21^g^ | 3.28±0.18^b^ | 3.58±0.16^a^ | 2.85±0.14^c^ |

Data are expressed as the mean ± standard deviation from replicate analyses (n=3) of three replicate samples. The different lowercase letters in each row indicate significant differences between samples (*P*<0.05).

# **Supplementary Table S3** Content of the aroma components in the Cabernet Sauvignon wine samples.

| Compound | RI | Threshold  (μg/L) | Concentrations(μg/L) | | | | | | | | | | | | |
| --- | --- | --- | --- | --- | --- | --- | --- | --- | --- | --- | --- | --- | --- | --- | --- |
|  |  |  | CK | | C1:1 | C1:5 | | | C1:10 | | S1:1 | | S1:5 | S1:10 | |
| **Esters** |  |  |  |  | | |  |  | |  | |  | | |  |
| Ethyl acetate | 885 | 5 | 4620±127^d^ | 4371±78^e^ | | | 4781±119^c^ | 7017±261^a^ | | 4967±96^c^ | | 5924±118^b^ | | | 7262±249^a^ |
| Ethyl butyrate | 1026 | 0.18 | 110±3^d^ | 108±15^d^ | | | 119±7^b^ | 117±21^b^ | | 113±11^c^ | | 130±9^a^ | | | 128±12^a^ |
| Isoamyl acetate | 1132 | 30 | 174±18^d^ | 166±14^e^ | | | 173±20^d^ | 243±26^b^ | | 157±19^f^ | | 194±24^c^ | | | 495±51^a^ |
| Hexyl formate | 1139 | NF | 72±11^f^ | 264±75^c^ | | | 218±64^d^ | 482±99^a^ | | 201±69^e^ | | 468±101^b^ | | | 452±88^b^ |
| Ethyl hexanoate | 1244 | 2.2 | 75±11^e^ | 69±8^e^ | | | 93±10^d^ | 292±31^b^ | | 117±12^c^ | | 341±31^a^ | | | 322±29^ab^ |
| Ethyl heptanoate | 1317 | 2 | 5±0^b^ | 2±0^e^ | | | 7±0^a^ | 7±0^a^ | | 4±0^d^ | | 6±2^ab^ | | | 7±1^a^ |
| Ethyl lactate | 1363 | 154636 | 23±4^d^ | 31±6^c^ | | | 22±3^d^ | 46±7^b^ | | 25±3^d^ | | 59±9^a^ | | | 54±9^a^ |
| Ethyl octanoate | 1446 | 15 | 807±112^g^ | 1492±126^e^ | | | 1846±171^c^ | 2105±199^a^ | | 1054±109^f^ | | 1612±154^d^ | | | 1997±213^b^ |
| Propyl lactate | 1472 | NF | 2811±93^e^ | 2643±139^f^ | | | 3223±108^d^ | 5883±337^b^ | | 6079±247^a^ | | 5256±150^c^ | | | 5728±152^b^ |
| Ethyl nonanoate | 1581 | 12000 | 1±0^b^ | 1±0^b^ | | | 1±0^b^ | 1±0^b^ | | 1±0^b^ | | 2±0^a^ | | | 1±0^b^ |
| Methyl decanoate | 1628 | NF | 1±0^c^ | 1±0^c^ | | | 1±0^c^ | 3±0^a^ | | 1±0^c^ | | 2±0^b^ | | | 2±1^b^ |
| Ethyl decanoate | 1651 | 23 | 315±29^d^ | 319±30^d^ | | | 339±26^c^ | 390±41^b^ | | 346±31^c^ | | 392±22^b^ | | | 426±44^a^ |
| Ethyl dec-9-enoate | 1712 | 100 | 46±6^c^ | 34±5^d^ | | | 51±9^b^ | 45±7^c^ | | 21±4^e^ | | 37±8^cd^ | | | 66±15^a^ |
| Methyl Salicylate | 1803 | NF | 5±0^e^ | 11±0^b^ | | | 7±0^d^ | 9±1^c^ | | 4±0^e^ | | 13±1^a^ | | | 11±1^b^ |
| Diethyl succinate | 1809 | 6000 | 11±1^b^ | 8±0^c^ | | | 8±1^c^ | 11±1^b^ | | 9±0^c^ | | 15±3^a^ | | | 11±2^b^ |
| Isoamyl Caprylate | 1826 | NF | 70±11^a^ | 17±2^d^ | | | 22±3^c^ | 31±6^ab^ | | 20±2^c^ | | 33±5^ab^ | | | 27±3^b^ |
| Ethyl laurate | 1864 | 1500 | 44±5^b^ | 46±6^ab^ | | | 37±4^d^ | 62±5^a^ | | 51±6^b^ | | 42±4^c^ | | | 39±4^d^ |
| Isoamyl decanoate | 1993 | NF | 6±0^c^ | 3±0^d^ | | | 1±0^e^ | 9±0^b^ | | 5±0^c^ | | 11±1^a^ | | | 8±0^b^ |
| Ethyl myristate | 2038 | NF | 7±0^cd^ | 6±0^d^ | | | 13±1^b^ | 15±1^ab^ | | 8±0^c^ | | 17±2^a^ | | | 12±1^b^ |
| Ethyl palmitate | 2248 | 1500 | 16±2^b^ | 4±0^e^ | | | 11±1^d^ | 14±2^c^ | | 15±1^b^ | | 21±2^a^ | | | 13±1^c^ |
| **Alcohols** |  |  |  |  | | |  |  | |  | |  | | |  |
| isopropyl alcohol | 1046 | NF | 41±6^d^ | 38±5^d^ | | | 46±7^c^ | 61±10^b^ | | 65±11^b^ | | 45±8^c^ | | | 87±11^a^ |
| Isobutyl alcohol | 1108 | 40000 | 194±20^e^ | 201±19^d^ | | | 214±26^c^ | 250±23^b^ | | 208±30^c^ | | 277±21^a^ | | | 285±23^a^ |
| Isoamyl alcohol | 1230 | 30000 | 70416±1486^d^ | 69130±1388^e^ | | | 91655±1029^c^ | 99474±1948^b^ | | 72678±1424^d^ | | 90447±897^c^ | | | 102853±1259^a^ |
| 2,3-Butanediol | 1284 | 150000 | 26±4^c^ | 21±4^d^ | | | 20±2^d^ | 29±4^b^ | | 21±4^d^ | | 32±5^a^ | | | 30±5^ab^ |
| 1-Hexanol | 1392 | 1100 | 392±26^f^ | 381±35^g^ | | | 403±29^e^ | 493±40^b^ | | 443±38^c^ | | 425±44^d^ | | | 533±61^a^ |
| 1-Heptanol | 1450 | 200 | 124±21^b^ | 55±9^e^ | | | 79±13^c^ | 121±25^b^ | | 67±11^d^ | | 119±22^b^ | | | 138±36^a^ |
| 1-Octanol | 1550 | 120 | 8±1^d^ | 8±0^d^ | | | 8±1^d^ | 12±2^b^ | | 9±0^c^ | | 12±2^b^ | | | 15±3^a^ |
| 2-Ethyl-4-methyl-1-pentanol | 1586 | NF | 7±0^d^ | 8±1^d^ | | | 12±2^b^ | 17±4^ab^ | | 10±2^c^ | | 25±8^a^ | | | 14±2^b^ |
| 2-Nonanol | 1605 | NF | 3±0^c^ | 1±0^e^ | | | 2±0^d^ | 4±2^b^ | | 3±1^c^ | | 2±1^d^ | | | 5±0^a^ |
| 1-Decanol | 1781 | 400 | 10±3^c^ | 7±0^d^ | | | 9±1^c^ | 15±3^a^ | | 11±0^bc^ | | 12±2^b^ | | | 14±2^a^ |
| Heptadecan-9-ol | 2470 | NF | 75±14^a^ | 48±10^c^ | | | 45±7^c^ | 72±13^a^ | | 59±11^b^ | | 63±11^ab^ | | | 56±9^b^ |
| A**cids** |  |  |  |  | | |  |  | |  | |  | | |  |
| Acetic acid | 1432 | 200000 | 775±26^e^ | 779±52^e^ | | | 902±66^c^ | 948±101^b^ | | 782±42^e^ | | 887±37^d^ | | | 981±59^a^ |
| Isobutyric acid | 1618 | 2300 | 21±2^ab^ | 26±3^a^ | | | 21±3^ab^ | 18±3^d^ | | 20±2^b^ | | 21±4^ab^ | | | 19±2^c^ |
| Hexanoic acid | 1863 | 420 | 1017±126^e^ | 948±102^f^ | | | 1547±153^a^ | 1468±152^b^ | | 1335±147^c^ | | 1194±109^d^ | | | 1586±148^a^ |
| Octanoic acid | 2083 | 500 | 5551±122^b^ | 4927±241^e^ | | | 5176±182^d^ | 5752±98^a^ | | 5261±157^c^ | | 4593±137^f^ | | | 5262±92^c^ |
| Decanoic acid | 2296 | 1000 | 831±58^d^ | 721±78^e^ | | | 694±55^f^ | 1012±124^a^ | | 874±62^c^ | | 828±94^d^ | | | 983±110^b^ |
| 9-Decenoic acid | 2356 | NF | 23±2^c^ | 16±1^e^ | | | 25±3^b^ | 29±2^ab^ | | 19±2^d^ | | 26±3^b^ | | | 31±3^a^ |
| Lauric acid | 2492 | NF | 9±0^d^ | 8±0^e^ | | | 12±1^b^ | 9±0^d^ | | 8±1^e^ | | 10±1^c^ | | | 15±2^a^ |
| **Phenylethyls** |  |  |  |  | | |  |  | |  | |  | | |  |
| Ethyl benzoate | 1640 | NF | 31±6^g^ | 34±6^f^ | | | 38±4^e^ | 59±7^b^ | | 43±5^d^ | | 52±6^c^ | | | 68±9^a^ |
| Phenethyl acetate | 1829 | 250 | 241±37^e^ | 192±21^f^ | | | 314±31^c^ | 483±44^b^ | | 269±25^d^ | | 304±29^c^ | | | 573±62^a^ |
| Benzyl alcohol | 1896 | 2000 | 1426±129e | 1481±137d | | | 1409±121f | 1696±119a | | 1709±151a | | 1506±144c | | | 1684±152b |
| Phenylethanol | 1931 | 14000 | 60986±297^g^ | 63706±204^f^ | | | 76857±362^d^ | 81210±472^b^ | | 74343±265^e^ | | 79608±358^c^ | | | 82848±408^a^ |
| **Terpenes** |  |  |  |  | | |  |  | |  | |  | | |  |
| α-Terpineol | 1706 | 1.2 | 42±5^e^ | 38±2^f^ | | | 42±4^e^ | 53±5^c^ | | 49±4^d^ | | 55±5^b^ | | | 59±3^a^ |
| β-Damascenone | 1832 | 0.05 | 11±4^c^ | 9±1^d^ | | | 14±4^b^ | 21±2^a^ | | 9±0^d^ | | 19±5^a^ | | | 16±2^b^ |
| citronellol | 1869 | 100 | 9±0^e^ | 16±2^d^ | | | 25±3^b^ | 29±3^a^ | | 11±1^e^ | | 21±2^c^ | | | 23±3^c^ |
| **Other compound** |  |  |  |  | | |  |  | |  | |  | | |  |
| 3,5-Di-tert-butylphenol | 2563 | NF | 15±1^c^ | 8±0^e^ | | | 13±2^d^ | 21±1^a^ | | 18±1^b^ | | 21±1^a^ | | | 20±2^a^ |

Data are expressed as the mean ± standard deviation from replicate analyses (n=3) of three replicate samples. The different lowercase letters in each row indicate significant differences between samples (*P*<0.05). Odor threshold in aqueous ethanol solution. The values were referred from http://www.leffingwell.com/odorthre.htm.

Supplementary Figures


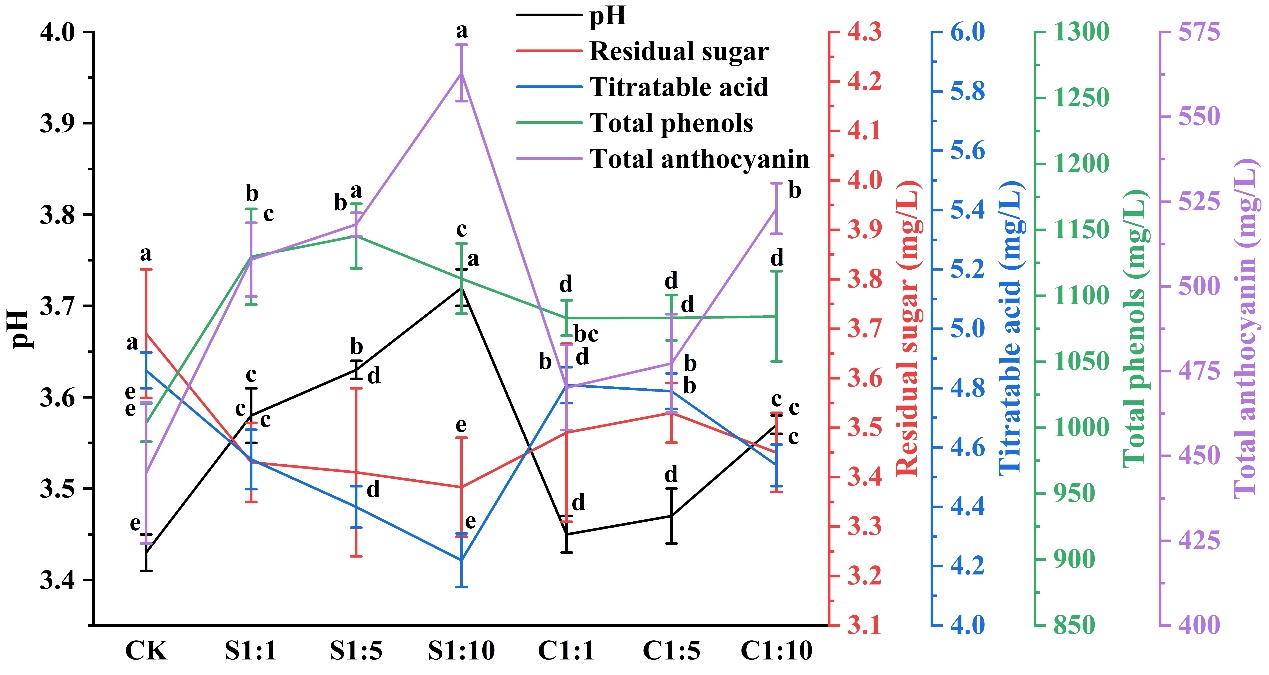


**Fig. S1** Effects of different fermentation methods on physical and chemical indexes of wine. pH (black), residual sugar (red), titratable acid (blue) and total phenols (green), total anthocyanin (purple).


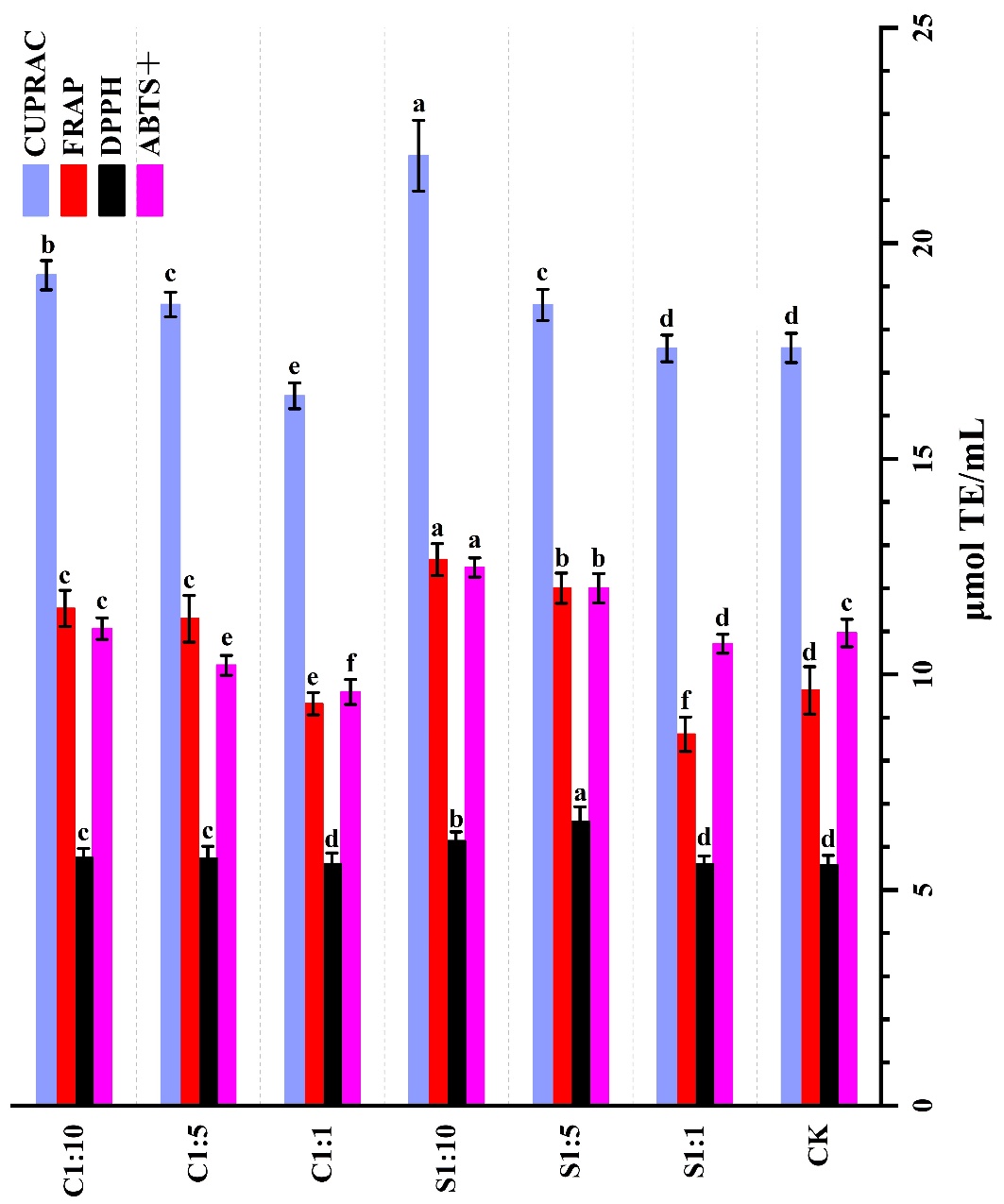


**Fig. S2** Effects of different mixed fermentation methods on four antioxidant activities of wine.
